# Coagulant activity of recombinant human factor VII produced by lentiviral human *F7* gene transfer in immortalized hepatocyte-like cell line

**DOI:** 10.1371/journal.pone.0220825

**Published:** 2019-08-05

**Authors:** Sarai Pongjantarasatian, Praguywan Kadegasem, Werasak Sasanakul, Khanit Sa-ngiamsuntorn, Suparerk Borwornpinyo, Nongnuch Sirachainan, Ampaiwan Chuansumrit, Pansakorn Tanratana, Suradej Hongeng

**Affiliations:** 1 Molecular Medicine Program, Faculty of Science, Mahidol University, Bangkok, Thailand; 2 Division of Hematology-Oncology, Department of Pediatrics, Faculty of Medicine, Ramathibodi Hospital, Mahidol University, Bangkok, Thailand; 3 Department of Biochemistry, Faculty of Pharmacy, Mahidol University, Bangkok, Thailand; 4 Department of Biotechnology, Faculty of Science, Mahidol University, Bangkok, Thailand; 5 Excellent Center for Drug Discovery, Faculty of Science, Mahidol University, Bangkok, Thailand; 6 Department of Pharmacology, Faculty of Science, Mahidol University, Bangkok, Thailand; Wake Forest Institute for Regenerative Medicine, UNITED STATES

## Abstract

Human mesenchymal stem cells (hMSCs) have the potential to differentiate into hepatocyte-like cells, indicating that these cells may be the new target cell of interest to produce biopharmaceuticals. Our group recently established a hMSC-derived immortalized hepatocyte-like cell line (imHC) that demonstrates several liver-specific phenotypes. However, the ability of imHC to produce coagulation factors has not been characterized. Here, we examined the potential for imHC as a source of coagulation protein production by investigating the ability of imHC to produce human factor VII (FVII) using a lentiviral transduction system. Our results showed that imHC secreted a low amount of FVII (~22 ng/mL) into culture supernatant. Moreover, FVII from the transduced imHC (0.11 ± 0.005 IU/mL) demonstrated a similar coagulant activity compared with FVII from transduced HEK293T cells (0.12 ± 0.004 IU/mL) as determined by chromogenic assay. We demonstrate for the first time, to the best of our knowledge, that imHC produced FVII, albeit at a low level, indicating the unique characteristic of hepatocytes. Our study suggests the possibility of using imHC for the production of coagulation proteins.

## Introduction

Coagulation factor VII (FVII) is a vitamin K‐dependent serine protease that is exclusively synthesized by liver. Human FVII is encoded by the *F7* gene on chromosome 13 at 13q34. Mature FVII has a molecular weight of approximately 50 kDa [1] and circulates in plasma in two different forms, the inactive zymogen and the active protease (FVIIa). The inactive zymogen is the major form of FVII and circulates at a concentration of 500 ng/mL, while FVIIa circulates at a significantly lower concentration (~0.5–5.5 ng/mL) [2, 3].

The recombinant activated FVII (rFVIIa) is commonly used for treatment in patients with hemophilia with inhibitors, and FVII deficiency [4–7]. The rFVIIa is produced by baby hamster kidney (BHK) cells and represents a recombinant protein derived from a mammalian cell line that is successfully used for therapeutic purposes. Some studies have highlighted a concern in using non-human cell lines because they produce proteins with glycosylation pattern different from those observed in human cell lines, as this may have a critical impact on the quality and functionality of recombinant proteins [8, 9]. In addition, one report showed that recombinant FVII (rFVII) produced from a human hepatic cell line (SK-Hep-1) was more active than rFVII from BHK-21 cells [9]. These studies indicate that identification of novel human cell lines that can be used for recombinant protein production is critical.

Human mesenchymal stem cells (hMSCs) have been recently established as a new tool for delivering secreted proteins because they are easy to isolate, expand, and manipulate with genetic modification [10]. Several studies showed that hMSCs derived from adipose tissue, bone marrow or umbilical cord blood are capable of differentiating into hepatocyte-like cells in specialized *in vitro* culture conditions [11–13]. hMSCs were immortalized using a combination of viral oncogenes (e.g. Bmi-1) and the human telomerase reverse transcriptase protein [14, 15]. This strategy has been successful in establishment of immortalized hepatocytes [16–21]. The differentiated hepatocyte-like cells derived from immortalized hMSCs demonstrate hepatocyte phenotypes, as determined by liver-specific markers such as drug-metabolizing enzymes. Our group previously successfully established a hMSC-derived-immortalized hepatocyte-like cell line (imHC) confirmed by hepatocyte markers and CYP450 isotypes [22]. Moreover, the imHC showed high induction of CYP450 transcripts. These results suggested that the imHC has the potential to serve as an alternative cell-based model for screening toxicity in clinical drug development. However, the functional capacity of these hepatocyte-like cells derived from hMSCs to produce coagulation factors has not been characterized.

Here we investigated the ability of the imHC to produce FVII by transduction of a lentivirus expressing the human *F7* gene under control of the CMV promoter. Our results showed that the imHC successfully produced a detectable level of FVII. Furthermore, FVII produced by the imHC was biologically active with coagulant activities that were comparable to FVII produced in HEK293T cells. Together these findings indicate that the imHC could be used as a cell source for the production of rFVII.

## Materials and methods

### Cells and cell culture

HEK293T and HepG2 cells were obtained from the American Type Culture Collection (ATCC). The imHC was developed as previously described [14]. HEK293T cells were grown in Dulbecco's Modified Eagle Medium (DMEM) high glucose containing 10% fetal bovine serum (FBS) (v/v), 2 mM L-glutamine, 100 units/mL penicillin, 100 μg/mL streptomycin and 110 mg/L sodium pyruvate. HepG2 cells and the imHC were grown in DMEM/F12 with 10% (v/v) FBS, 2 mM L-glutamine, 100 units/mL penicillin and 100 μg/mL streptomycin. All cells were grown in monolayer in 37°C with 5% CO_2_ and 95% humidity. Culture media were changed every three days.

### Construction of lentivirus expressing FVII

We generated a lentivirus containing human FVII cDNA expressed under the control of the CMV promoter. RNA extracted from HepG2 cells was converted into cDNA, which was then used as template for human FVII cDNA synthesis. The full-length coding sequence of human FVII was PCR amplified using specific primers flanked with XhoI and BamHI sites as follows: hcDNA/FVII-XhoI-**KoZ**-F: 5′- TT CTC GAG**GCC ACC** ATG GTC TCC CAG GCC CTC AGG CTC CT-3′, and hcDNA/FVII-BamHI-R: 5′- GG GGA TCC CTA GGG AAA TGG GGC TCG CAG GA-3′ (restriction sites are shown in underlined text). The 1,357 bp PCR product size of human FVII was verified on an agarose gel stained with 1% GelRed (Biotium, Hayward, CA, USA) and purified using a PCR purification kit (QIAGEN, Valencia, CA, USA). The purified PCR product was ligated into the pTG19-T PCR cloning vector (Vivantis Technologies, Sdn Bhd, Malaysia). The correct clone was confirmed by direct sequencing (Macrogen Inc., Seoul, Korea), and the human FVII cDNA was subsequently subcloned into the pLVX-puro vector (FVII vector) (Clonetech Laboratories, CA, USA), which was further confirmed by sequencing.

### Generation of lentiviral particles and assessment of viral titer

Lentivirus encoding FVII was generated by transient co-transfection of HEK293T cells using a second-generation packaging system. Briefly, non-confluent HEK293T cells in 10-cm culture dishes were transfected with FVII vector, pSPAX2 (packaging plasmid; gag/pol, Addgene (Watertown, MA), plasmid #12260), and pMD2.G (envelope plasmid; VSV-G, Addgene, plasmid #12259) by the CaPO_4_-DNA co-precipitation method [23]. Lentiviruses were harvested at 72 h post-transfection, concentrated by a Lenti-X concentrator (Clonetech Laboratories) and stored at -70 ˚C in single-use aliquots. Similarly, a negative control virus (empty vector) was produced by co-transfection of HEK293T cells with pLVx-puro vector, pSPAX2, and pMD2.G.

The infectious titer was determined in HEK 293T cells by plaque assay using puromycin selection. Briefly, 2 x 10^5^ cells were seeded in 6-well plates prior to infection. Serial dilutions of lentiviral stocks (10-fold dilution up to 6 times) were prepared in HEK 293T culture medium. HEK 293T cells were incubated with 1 mL of viral dilution for 24 h at 37°C before the viral suspension was aspirated and replaced with 2 mL of fresh culture medium. At 48 h- post infection, medium was replaced with 2 mL of HEK 293T culture medium containing puromycin (2 μg/mL; Sigma Aldrich). Puromycin selection was applied for 11 days, during which puromycin-containing medium was replenished every 3 days. Colonies were stained with 1% crystal violet (Sigma-Aldrich) and counted. Viral titers were calculated from wells containing between 20 and 100 colonies by the following formula: (number of discrete colonies x dilution factor)/volume of inoculum.

### Lentiviral transduction and expression of FVII

Cell lines (imHC, HepG2 and HEK293T) were transduced with lentivirus at multiplicity of infection (MOI) of 10 in culture media containing 8 μg/mL polybrene. Spinoculation was used for increasing transduction efficiency by centrifugation at 800 g, 32°C for 90 min. At 72 h later, transduced cells were selected with 2 μg/mL puromycin for 2 weeks. Puromycin-resistant cells were then expanded in growth medium containing 10 μg/mL vitamin K1 (Roche, Basel, Switzerland).

Transduced cells were seeded at density of 3x10^6^ cells and maintained in growth medium containing 10% FBS and 10 μg/mL vitamin K1 for 3 days. Next, cells were serum-starved for 2 days in growth medium (without FBS) supplemented with 1x insulin-transferrin-selenium (EMD Millipore) and 10 μg/mL vitamin K1. Cultured media were collected and centrifuged at 3,000 x g for 10 min at 4°C to remove cell debris. Collected media were combined with benzamidine and PEG-8000 at final concentration of 10 nM and 0.1%, respectively, and then concentrated using 30 kDa molecular weight cut-off membranes (Amicon Ultra Centrifugal Filter Devices, Millipore, Carrigtwohill, Country Cork, Ireland) [24, 25]. Not more than 5 passages of transduced cells were used in all experiments after puromycin selection process was completely done.

### RNA extraction and real-time PCR

Total RNA was extracted from non-transduced and transduced cells using the RNeasy Mini Kit (QIAGEN, Hilden, Germany) following the manufacturer’s instructions. Genomic DNA contamination was eliminated by DNase I, and the RNA concentration was measured by UV spectrometry. One microgram of total RNA was converted into cDNA with the Superscript III First-Strand Synthesis System for RT-PCR kit (Invitrogen, USA) according to standard protocol. Relative gene expression profiles were determined by real-time PCR based on SYBR green detection with the *F7* gene specific primers. The ΔCT was calculated by subtraction the raw CT values of *F7* gene with glyceraldehyde-3-phosphate dehydrogenase (GAPDH). Data were presented as 2^-ΔCT^.

### Measurement of FVII concentration in culture media

To determine the level of secreted FVII in cell culture supernatant, transduced cells were cultured in serum-free media for 48 h in static culture condition. Culture media were harvested from the transduced cell lines when cells were 80% confluent. The level of FVII in culture media was determined using the Human Factor VII ELISA Kit (Abcam, Cambridge, UK) according to the manufacturer’s instructions.

### Determination of FVII coagulant activity

FVII activity was measured by chromogenic and clotting assays. For chromogenic assay, FVII-containing cultured media were examined using the Factor VII Human Chromogenic Activity Kit (Abcam) according to the manufacturer’s instructions. FVII from each sample cell line was diluted to 0.0134 nM (0.067 ng/mL) in sample diluent and analyzed in duplicate. A standard curve was established by 1.5-fold serial dilution to achieve 8 standard points of human FVII standard protein (0.15 IU/ml, 0.1 IU/ml, 0.067 IU/ml, 0.044 IU/ml, 0.0296 IU/ml, 0.0198 IU/ml, 0.0132 IU/ml and 0 IU/ml = blank diluent sample control) in sample diluent and run in duplicate. All evaluations were performed in three independent experiments (n = 3). For chromogenic assay, 0.01 nM FVII was employed in all experiments.

For clotting assays, the coagulant activity of FVII was determined by modified prothrombin time in lyophilized FVII-deficient plasma (Siemens, Midrand, South Africa) using human recombinant thromboplastin (RecombiPlasTin 2G, HemosIL) as the activator. FVII samples were diluted to 0.5 nM (25 ng/mL) in FVII-deficient plasma. The assay was performed on the ACL 200 (Instrumentation Laboratory, Bedford, MA, USA).

### Western blot analysis

The total protein concentration from concentrated cultured supernatant was measured by Bradford protein assay. Concentrated proteins were mixed with disulfide-reducing Laemmli buffer and boiled at 98°C for 5 min. Protein samples were run on a 12% bis-tris SDS-PAGE and transferred to a PVDF membrane (Thermo Fisher Scientific, USA). Membranes were blocked overnight with 5% skimmed milk in Tris-buffered saline with 0.1% Tween-20 (TBS-T). The blocked membranes were washed three times with TBS-T. Next, the membranes were incubated with 1:10,000 dilution of monoclonal rabbit anti-human FVII (Abcam) for 3 h at room temperature. Membranes were washed three times with TBS-T and then incubated with 1:20,000 dilution of peroxidase-conjugated goat anti-rabbit IgG H&L (Abcam). The blot was developed using the ECL Prime Western Blotting System (Amersham, UK) and visualized by X-ray film exposure.

### Statistical analysis

Data were expressed as mean ± SEM from three independent experiments (n = 3). FVII activity between two cell lines was compared using Student’s t-test or a one-way ANOVA followed by Tukey’s multiple comparison test. Statistical significance was considered at *p* < 0.05. All statistical analyses were carried out by GraphPad Prism 7 (GraphPad Software Inc., La Jolla, CA, USA).

## Results

### FVII mRNA expression is upregulated in cell lines transduced with human FVII-expressing lentivirus

**Fig 1:**
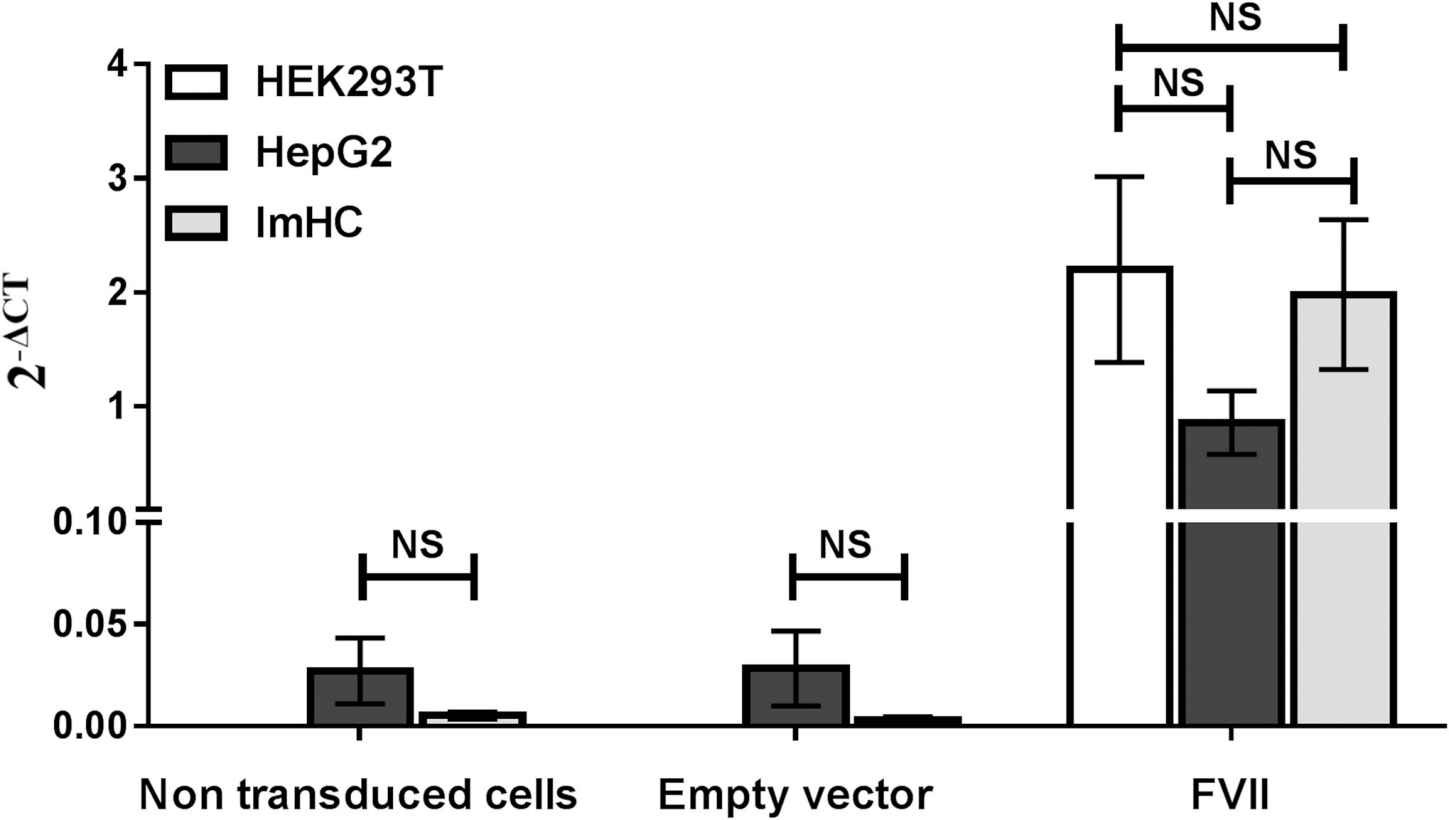
FVII mRNA expression in cell lines after transduction with lentivirus. Cell lines were transduced with the indicated lentivirus and the expression (2^-ΔCT^) of *F7* gene was determined and normalized with the endogenous housekeeping gene, glyceraldehyde-3-phosphate dehydrogenase (*GAPDH*). Data are shown as mean ± SEM from three independent experiments. There was no statistical difference in FVII expression among cell lines (one-way ANOVA test). NS; not significant.

We transduced lentivirus expressing human FVII (encoded by the *F7* gene) in the imHC, HEK293T cells and HepG2 cells and examined FVII mRNA expression levels in all three cell lines (Fig 1). Non-transduced cells and cells transduced with empty vector were used as the negative controls. FVII mRNA levels in all cell lines transduced with empty vector were similar to those in non-transduced cells, and non-transduced HEK293T did not express detectable levels of FVII mRNA. FVII mRNA levels were increased in HepG2 and imHC infected with human FVII-expressing lentivirus by approximately 31.77-fold and 372.45-fold, respectively, compared with non-transduced cells. Similarly, increase of FVII mRNA expression was observed in HEK293T cells transduced with FVII-expressing lentivirus.

### Cell lines transduced with FVII-expressing lentivirus secreted FVII in cell culture supernatant

**Fig 2:**
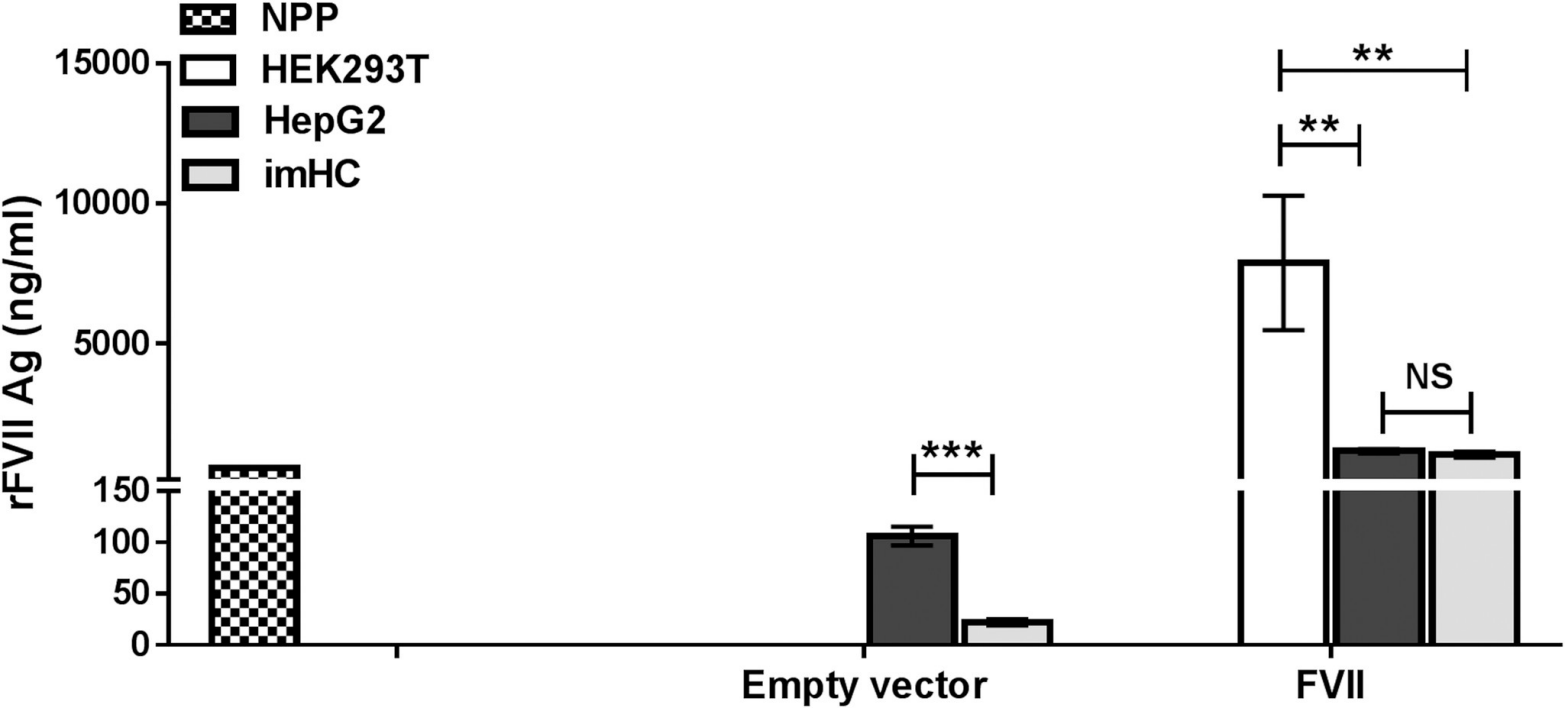
FVII levels secreted in culture media supernatant. Cell lines were transduced with the indicated lentivirus, and FVII levels in culture supernatant were determined by ELISA. Normal pooled plasma (NPP) was used as a positive control. Data are reported as mean ± SEM (n = 3). There was a significant difference in FVII level between Empty vector- HepG2 vs Empty vector- imHC (p = 0.0005), FVII-HEK293T vs FVII-HepG2 (p = 0.0042), and FVII-HEK293T vs FVII-imHC (p = 0.0036). No significant difference was observed in FVII-HepG2 vs FVII-imHC (p >0.9999). Statistical differences were analyzed with paired student *t*-test for Empty vector- HepG2 vs Empty vector- imHC and one-way ANOVA test for FVII-HEK293T vs FVII-HepG2, and FVII-HEK293T vs FVII-imHC (p = 0.0036). **; p<0.01, ***; p<0.001.

We next examined FVII levels in culture supernatant from the transduced cell lines using ELISA (Fig 2). Normal pooled human plasma (NPP) was used as a positive control. The FVII level in NPP was 552.90 ± 11.94 ng/mL, which is within the physiologic range of circulating FVII concentration [2]. HEK293T cells transduced with empty vector showed no detectable FVII, which was consistent with the FVII mRNA results. No basal level of FVII expression in HEK293 was also reported in the Human Protein Atlas Database. FVII levels in HepG2 and imHC lines transduced with empty vector were 106.00 ± 9.08 ng/mL and 22.2 ± 3.18 ng/mL, respectively.

Among the cell lines transduced with the FVII lentivirus, HEK293T cells secreted the highest amount of FVII (7,873.00 ± 2398.46 ng/mL) compared with HepG2 (1,164.00 ± 75.81 ng/mL) and imHC (1,039.00 ± 108.38 ng/mL). This result demonstrated that imHC was capable of producing comparable amounts of FVII compared with HepG2 cells.

### FVII secreted from cell lines transduced with FVII-expressing lentivirus exert coagulant activity

We next evaluated the biological activity of secreted FVII in culture media supernatant using chromogenic and one-stage clotting assays. Each assay was performed using equal amounts of FVII as determined by ELISA.

**Fig 3:**
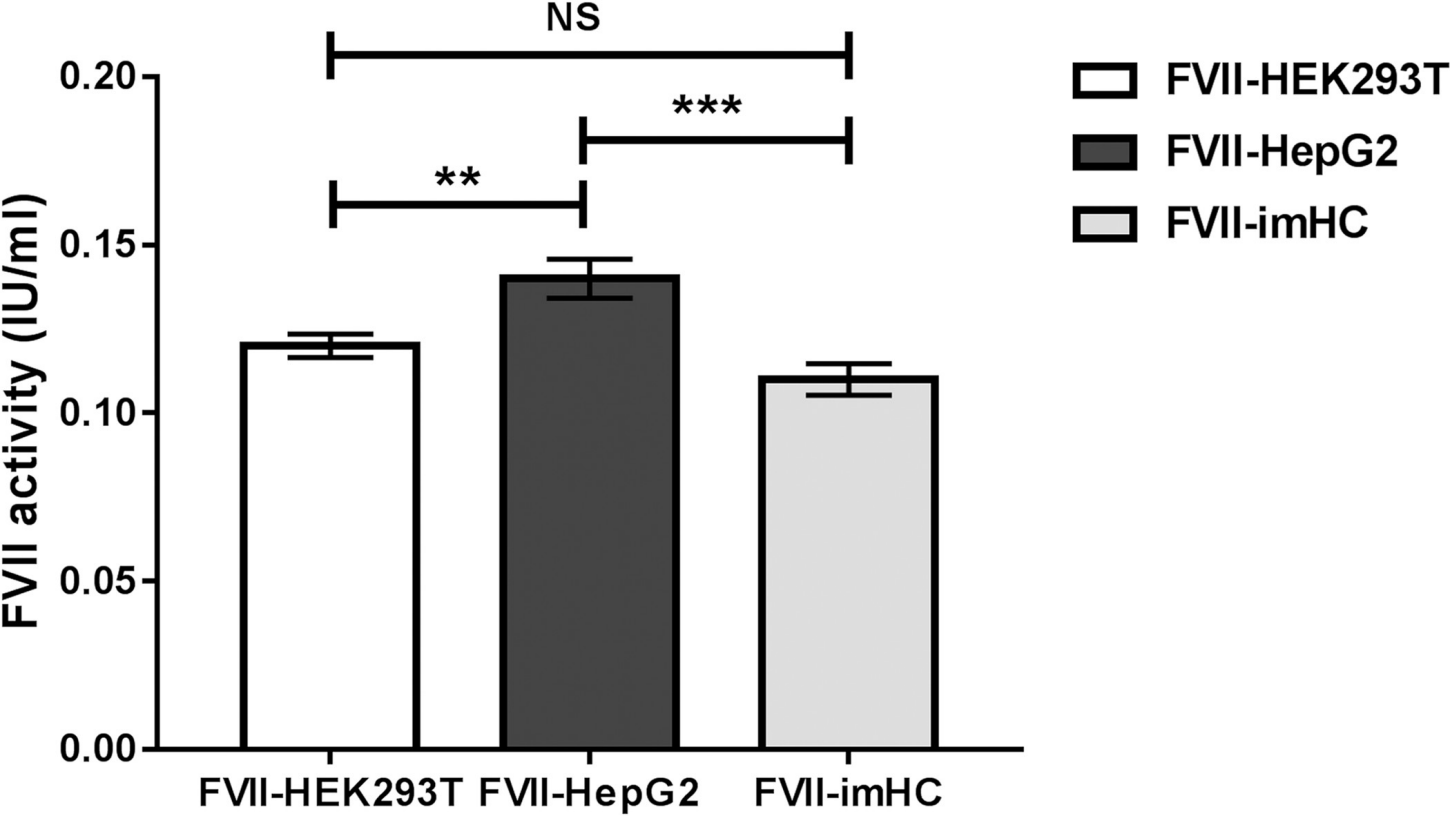
FVII coagulant activity from each cell line determined by chromogenic assay. FVII coagulant activity was determined by the chromogenic assay (two-step FXa generation). Equivalent molar concentrations (0.01 nM) of FVII from each cell line were used to evaluate coagulant activity, which was reported as IU/mL (mean ± SEM). The coagulant activity between two cell lines was compared using Student’s t-test. **p < 0.05. NS; not significant.

FVII produced from transduced HepG2 cells (FVII-HepG2) demonstrated the highest FVII activity (0.14 ± 0.006 IU/mL) (Fig 3). There was no significant difference in FVII activity between FVII produced from transduced HEK293T cells (FVII-HEK293T, 0.12 ± 0.004 IU/mL) and FVII produced from the transduced imHC (FVII-imHC, 0.11 ± 0.005 IU/mL) (*p* = 0.1655). However, these results were not consistent with those of the one-stage clotting method, which measured time-to-clot after addition of FVII produced from each cell line in FVII-deficient plasma. FVII-HEK293T completely normalized the clotting time (10.4 sec compared with 71.6 sec without FVII), whereas the clotting time was still slightly prolonged with FVII-HepG2 (17.7 sec) and FVII-imHC (20.7 sec) n = 1 (Table 1).

**Table 1:** Coagulant activity of FVII determined by one-stage clotting assay.

Cell Line	Clotting Time (sec)
Empty vector-HEK293T FVII-HEK293T	71.6 10.4
FVII-HepG2	17.7
FVII-imHC	20.7

Empty vector-HEK293T represents HEK293T cells transduced with an empty vector (no *F7* gene) and serves as a negative control in the one-stage clotting assay. FVII-HEK293T, FVII-HepG2 and FVII-imHC represent FVII obtained from culture supernatant from cells transduced with *F7*-containing lentivirus.

### Different forms of FVII are detected from cell lines transduced with FVII-expressing lentivirus

**Fig 4:**
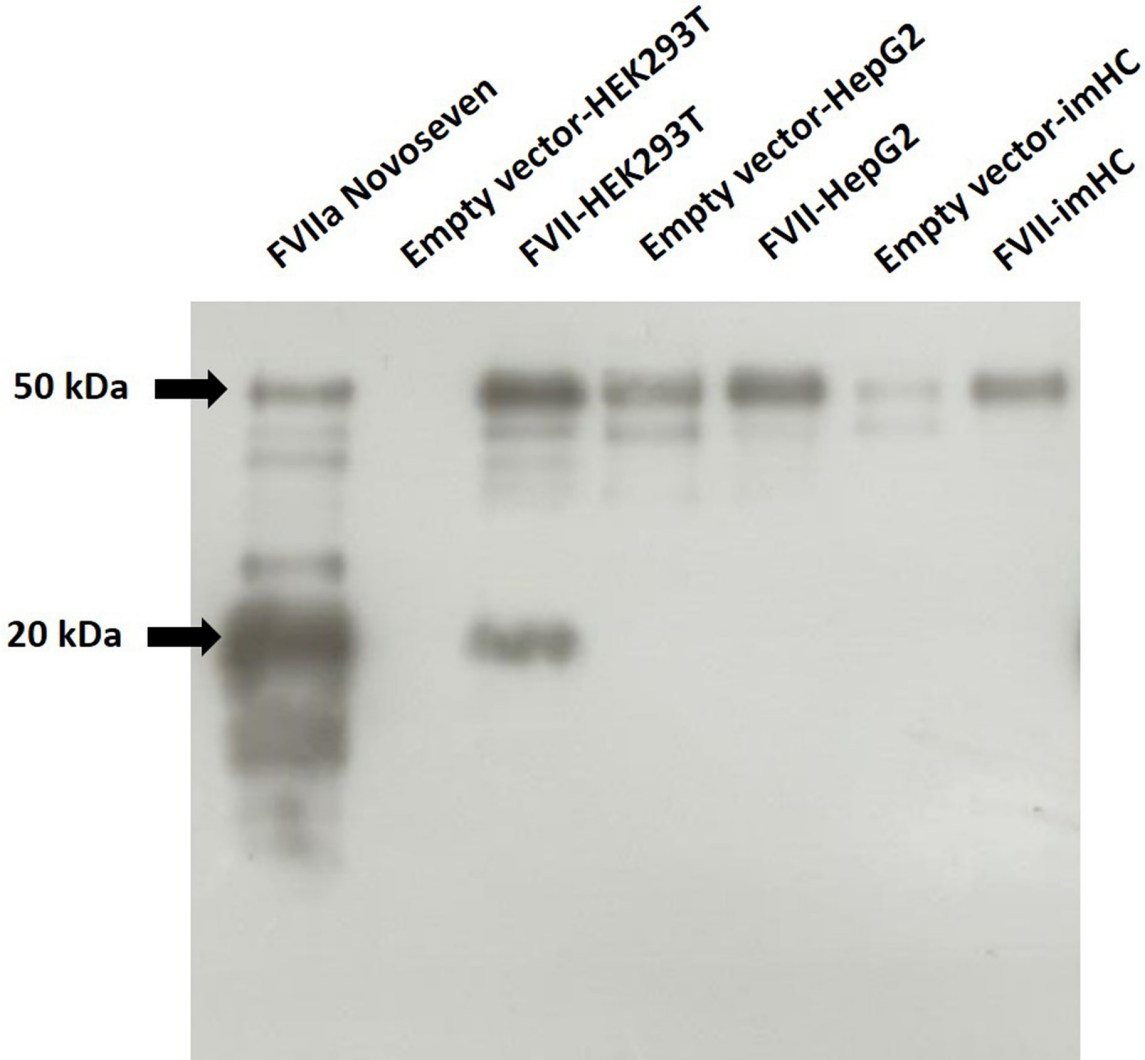
Western blot analysis of FVII in culture media supernatant. Western blot analysis of protein samples from the culture media supernatant from the indicated lanes (10 μg and 1 μg of total proteins were used for empty vector constructs and FVII constructs, respectively). Recombinant FVIIa (Novoseven, 500 ng) was used as a positive control. The zymogen FVII (MW ∼50 kDa) and the light chain of the protease FVIIa (MW∼20 kDa) are marked with arrows.

Because of the discrepancy of coagulant activities between chromogenic and one-stage clotting assays, western blot analysis under reducing condition was performed to verify the forms of secreted FVII. While the zymogen FVII consists of a single chain of approximately 50 kDa, the protease FVIIa consists of one light chain linked with one heavy chain by a single disulfide bond [2]. FVII-HEK293T demonstrated the mixture of zymogen FVII (MW∼50 kDa) and the light chain of protease FVIIa (MW∼20 kDa), whereas FVII-HepG2 and FVII-imHC showed only the zymogen form (Fig 4). Among cells transduced with empty vector, only HepG2 cells showed the zymogen FVII.

## Discussion

In this study, we examined the ability of the hMSC-derived imHC for the production of recombinant FVII by ectopic expression of human FVII using a lentiviral transduction system. Lentiviral vector for gene transfer was used because of the high capacity of lentivirus to transduce quiescent cells [26]. The result demonstrated that this system could efficiently transduce differentiated, non-dividing hepatocytes. HepG2 cells were used as a positive control and to evaluate the potential of FVII expression in imHC because HepG2 exerts several features of differentiated hepatocytes [27–30] and as these cells were previously demonstrate to express FVII [31–33]. HEK293T cells were also included as this is a commonly used human cell line for the production of biopharmaceuticals.

To best of our knowledge, this is the first study to demonstrate the functionality of imHC regarding the production of coagulation proteins. Not surprisingly, imHC expressed a very low amount of FVII mRNA, and the presence of FVII zymogen in culture supernatant from imHC was consistent with the mRNA results. The ability of non-transduced imHC to synthesize FVII is one of the characteristics of mature hepatocytes. These results together with our previous study [14, 22] suggest that the imHC functionally behaves like hepatocytes. Although the basal level of zymogen FVII in empty vector transduced-imHC was quite low, transduction with FVII-expressing lentivirus resulted in ~47-fold higher zymogen concentration. However, the FVII level from the transduced imHC was still markedly lower compared with FVII levels from transduced HEK293T cells.

We further evaluated biological FVII coagulant activity by chromogenic assay and found that FVII-HepG2 exerted the highest FVII activity compared with FVII-HEK293T and FVII-imHC-FVII. Notably, FVII-imHC showed similar coagulant activity as FVII-HEK293T. In contrast, one-stage clotting assay revealed that FVII-HEK293T had the best coagulant activity, followed by FVII-HepG2 and FVII-imHC. Western blot analysis of concentrated culture supernatant revealed a significant amount of FVIIa (biologically active form) from HEK293T cells but not from the HepG2 and imHC. The presence of FVIIa could be a result from FVII autoactivation occurring during the preparation process [34]. Therefore, the discrepancy of FVII coagulant activities obtained from chromogenic and one-stage clotting assays could be due to the presence of FVIIa in HEK293T cells.

The major difference between the two coagulant activity assays is the end-point. The modified one-stage clotting assay measures the time-to-clot in FVII-deficient plasma into which FVII(a) is added. This clotting time reflects the ability of FVII-deficient plasma to generate thrombin and ultimately insoluble fibrin in response to added FVII(a). In contrast, the chromogenic assay measures the capability of FVII(a) to generate factor Xa (FXa) in a buffer-based system. In the plasma-based clotting assay, in which tissue factor (TF) is already available, FVIIa can promptly form a TF-FVIIa complex on phospholipid membranes, resulting in the formation of insoluble fibrin clots [35]. However, zymogen FVII requires the activation to FVIIa prior to forming a complex with TF. Therefore, culture supernatant from HEK293T cells, which contained a high amount of FVIIa, demonstrated better FVII coagulant activity.

In contrast, the end-point of the chromogenic assay is the generation of FXa by the TF-FVIIa complex. The most critical enzymatic event in the chromogenic assay occurs during the 30-min incubation period among the reaction reagents (FVII(a), TF and FX). This period of incubation should provide ample time for the complete conversion of any FVII to FVIIa by the trace amount of generated FXa and TF-FVIIa complex [36]. Therefore, the FVII coagulant activity measured by this method primarily depends on the formation of FXa by the TF-FVIIa complex regardless of the initial form of FVII in the culture supernatant.

The addition of benzamidine in all buffers used during the FVII purification process is recommended to minimize the rate of FVII autoactivation [34, 37]. In our study, benzamidine was added at a final concentration of 10 nM during collection and concentration of FVII. However, after desalting, final FVII preparations were preserved in storage buffer without benzamidine (20 mM HEPES, 150 mM NaCl, pH 7.4). This could potentially lead to the generation of FVIIa in HEK293T cells and is a limitation of our study. Our results suggest that the chromogenic assay was more reliable for comparing the FVII coagulant activity among cell lines, particularly, in our study.

In conclusion, here we demonstrate for the first time the function of imHC in producing coagulation proteins. Our data confirmed a liver-specific phenotype of imHC as shown by the ability to synthesize a basal level of FVII. Moreover, the coagulant activity of FVII-imHC was quite similar to FVII-HEK293T in cells transduced with lentivirus. In terms of feasibility, the imHC is easy to grow and require only fundamental culture medium conditions. The doubling time of imHC is approximately 45 h [38], which is similar to that of HepG2. Our previous study showed that imHC was able to grow in a monolayer without sub-culturing and demonstrated contact inhibition after getting 100% confluency [38]. Together these results indicate the imHC may be useful in biopharmaceutical production especially in thrombosis and hemostasis research. Detailed PTM patterns and enzymatic activity assays of FVII-imHC should be further studied.
